# A Report of Fifteen Cases of Tracheotomy in Diphtheritic Croup, Six of Them Successful

**Published:** 1878-02

**Authors:** R. G. Bogue

**Affiliations:** Surgeon to Cook County Hospital; 3 Washington Place, Chicago


### A REPORT OF FIFTEEN CASES OF TRACHEOTOMY IN DIPHTHERITIC CROUP, SIX OF THEM SUCCESSFUL.

BY R. G. BOGUE, M. D., SURGEON TO COOK COUNTY HOSPITAL.

*(Read before the Chicago Medical Society.)*

The following cases are reported, not only for the general interest in themselves, but from the special interest which pertains to the subject; and because I believe that the profession needs more specific information upon the subject of tracheotomy for croup, and more encouragement to resort to the operation.

I am satisfied that there is a general want in the profession of a correct understanding of the disease—croup—and of what may and what may not be expected from medicine and the powers of nature to afford relief. Medical men do not, as a rule, appreciate the benefits to be derived from tracheotomy when employed as a remedial measure at the appropriate time, instead of as a " last resort," as is too generally taught.

As long as it is regarded a " last resort," after prolonged and fruitless trial of the usual remedies to relieve the larynx, continued until the child is about to die, there will be but little to hope for from the operation. But when it comes to be understood that medicines alone will not relieve the larynx in time to save the life of the patient, after respiration is embarrassed to a certain extent (exception being made of rare cases), and that the operation, as a rule, does not add gravity to the case, then very much may be expected from tracheotomy. Then, instead of one recovery in from five to ten cases, we believe there will be from fifty to seventy per cent. of successes, if the operation is resorted to reasonably early, and the cases carefully attended afterward. Cases of this sort invariably require a great deal of intelligent attention, especially for the first few days, and he who expects good results must be ready to devote himself to his little patients. If the study of the subjoined cases lead to fuller investigation of the subject, and assistance be derived from the few hints given as to the care of such patients, who thus may be given the benefit of an early, instead of a late operation, or none at all, the object of this paper will have been accomplished. We would refer to Trousseau's article on the subject, in his "Lectures on Clinical Medicine," and to Cohen's valuable little book on "Croup in Its Relations to Tracheotomy," for full details respecting the disease, the operation and the care and after-treatment of this class of patients.

CASE I.—Feb. 21st, 1874. Frankie Baxter, age 4 years. A healthy, well-nourished boy; coughed hoarsely on the 19th, but had no trouble during the night; 20th, coughed hoarsely, and breathing began to be difficult; these symptoms rapidly increased in intensity. About 5 o'clock, Dr. W. H. Byford saw him, directing the usual relaxing remedies, which produced nausea and even vomiting, but no relief to the respiration. At 6:30 I saw him with Dr. B. The respiration was then quite difficult, cough harsh and dry; the remedies were continued, and a solution of glycerine inhaled by means of the steam atomizer. We met again about 10 o'clock. The remedies had been continued faithfully, but without relief; the respiration had steadily become more difficult; both inspiration and expiration laborious; supra and infra-sternal spaces retracted markedly at each effort of inspiration. It seemed as though there was escape only by way of tracheotomy, as there had been steadily increasing embarrassment of respiration from the first, which had not been influenced in the least for the better by the remedies, and now it seemed impossible for the boy to live but a few hours if the obstruction continued to increase as it had during the several hours preceding. There was some cough and occasional spasm of the larynx, as at times there would be intervals of greater difficulty of respiration. About half-past 12, Dr. H. A. Johnson saw the patient with us, concurring in the opinion that the little boy could live but a few hours, unless relieved. He advised the operation without delay. Chloroform was given, and, with the assistance of Drs. Johnson and Byford, I performed tracheotomy, and introduced a tube. The patient breathed with perfect ease through the tube, and reacted from the anæsthetic readily. There was a good deal of cough and discharge through the tube of a tenacious mucus, which necessitated its frequent cleaning. The temperature of the room was kept at from 75° to 80° all of the time, for nearly two weeks, and the air kept moist by water boiling over the gas. For several days a solution of glycerine and chlorate of potass, in spray from the atomizer, was breathed almost constantly, and at frequent intervals afterward. For several days the cough was severe, and the discharge of mucus was very abundant, but this diminished gradually. The boy was fed milk, beef tea, coffee or tea with milk, as he preferred, and solid food as soon as he wanted it. The obstruction in the larynx continued for about one week, then gradually disappeared, so that at the end of the second week breathing through the larynx was quite free, and the tube removed the sixteenth day. The opening in the neck closed in a few days, the bronchitis soon disappeared, and there was no return of difficult breathing.

Case II.—Josie Moses, aged 4 years, a patient of Dr. Adolphus; sick about three weeks with diphtheria. The exudation and ulceration had been quite extensive, and the boy's strength reduced. I saw him about 11:30 p. m., on the 26th of March, 1874, on account of laryngeal complication, which began about twenty-four hours before, and had steadily increased in severity. When I saw him, the respiration was very imperfect and accomplished only by great effort; inspiration and expiration equally noisy and labored. The supra- and infra-sternal spaces thoroughly retracted during every effort at inspiration. It was very evident that the little boy could live but a few hours, if not relieved. The usual remedies having been judiciously used without benefit, it was decided to perform tracheotomy without delay. Everything was made ready, chloroform given, and, with the assistance of Drs. Adolphus, Hotz and Shaffer, I opened the trachea and introduced a tube about 1 a. m., 27th. After the discharge of a little bloody mucus through the tube, the child breathed very quietly, and reacted from the anæsthetic. The atmosphere of the room was raised to about 75°, and made moist by steam from boiling water. Also from the steam atomizer there was inhaled a solution of glycerine and chlorate of potassa. From the severity and extent of the diphtheritic disease of the fauces and at the top of the glottis, and consequent paralysis of the muscles of the larynx, œdema of the glottis and deformity of the epiglottis, respiration through the larynx was greatly delayed, so that the tube was not removed until the end of four months. After the introduction of the tube there was no more difficulty in breathing while carried on through the tube, but there was a tedious convalescence from the sickness. The boy, however, thoroughly recovered, the opening in the neck closing a short time after the removal of the tube.

Case III.—Brice Miller, aged 5 years, had been sick about one week with diphtheria. He was a patient of Dr. Hunt. I was called to see him at 4.30 a. m., Dec. 15, 1874, and found Dr. Freer with the little boy, Dr. Hunt being detained elsewhere. Laryngeal complication began some 24 or 36 hours before. When I came, the little patient was lying upon his back upon the bed, wholly unconscious, and merely gasping, perfectly flaccid. With the imperfect attempt at respiration, there was the harsh whistling sound in the larynx and depression of the sternal spaces. The boy seemed just about to die ; but I prepared a tube and immediately opened the trachea, and introduced it with the assistance of Dr. Freer. The child was so insensible, that he made no movement during the operation. Respiration very soon became pretty free, but presently there was evidence of obstruction in the tube, which was not overcome by passing through it a probe armed with cotton. The tubes were removed, when there appeared in the wound a large portion of membrane; this, with several small pieces, was removed, the tube replaced, when respiration became easy and efficient, and the little boy saved from his imminently dangerous condition. He soon made signs for some water, which he drank, also some milk. The temperature of the room was raised to 75°, and the air made moist by steam from boiling water. The operation was made about 5 a. m. All of that day and evening, up to 11 p. m., when I saw him, he had been entirely comfortable, taking fluid food freely, and breathing with perfect ease, and there had been discharged so much false membrane, that I felt quite easy about the case. Not thinking there was danger of acute obstruction, I left him for the night, with the direction that if the breathing became at all interfered with, to remove the inner tube and send for me. About 4.30 a. m., his friends noticed that he was not breathing quite as easily, and did as I had directed. I came very soon after 5 a. m., and found the boy dead. I removed the tube and passed a probe down the trachea, but felt no obstruction. It excited no movement, and there was no membrane in the tube. The boy had drunk a glass of milk, and sat on the chamber to urinate; very soon afterward, the breathing suddenly ceased. Up to this time there had been but very little interference with respiration. Did death occur from a large piece of membrane becoming loose and obstructing the bronchia, or was it from syncope? We believe the latter to have been the cause of the fatal result.

Case IV.—February 10th, 1876; Emma Kerga, aged 3 years, a patient of Drs. Church and Bert, had been ill of diphtheria somewhat over a week. I saw her on account of laryngeal complication, which first showed itself some 24 hours before, and had steadily increased until the breathing had become very difficult. When I saw her, there was very little air passing the point of obstruction in the larynx, and that only by great effort. The sternal spaces were drawn inward very markedly at every effort at inspiration. I advised an operation, and with the assistance of Drs. Church and Bert, opened the trachea, and introduced a tube. Chloroform was given. Breathing through the tube was quite easy; some small pieces of membrane were discharged; the temperature was kept at about 75°, and the air moistened by boiling water. Everything progressed well until about 24 hours after the operation, when the respiration became more hurried and the pulse faster. I saw the patient about 4½ p. m. The breathing was hurried, shallow and noisy from accumulation in the trachea. The child had lost strength since the day before. I removed the tubes; excited cough by passing a probe down the trachea; a few pieces of membrane and some mucus were discharged. The tube was replaced, but the respiration became more hurried, the pulse feebler, and the child died about 32 hours after the operation.

This patient died from what would be called extension of the disease into the deeper part of the bronchial passages, but the event was probably precipitated by an extensive pneumonic congestion or infiltration of the lung, which had begun before the operation.

CASE V.—April 29th, 1876; Seissman, aged 2½ years; a patient of Dr. Wadsworth; ill of diphtheria for several days; had extensive exudation in fauces and laryngeal complication, said by the parents to have commenced on the 28th. I saw the child about 9 a. m. The breathing was very inefficient and performed with a great deal of effort. I advised an operation, although it seemed as though it promised but little, in fact but temporary relief. A little chloroform was given, and with the assistance of Dr. Wadsworth, I opened the trachea and introduced a tube. When the trachea was first opened, there was expelled a quantity of thick muco-pus, which had accumulated in the trachea and larynx. Respiration was easier and without obstruction, but remained hurried. The child took nourishment, but gradually failed, and died about 24 hours after the operation—I believe from a commencing pneumonia.

CASE VI.—August 27, 1876; Larrey McMullen, age 5½ years, a patient of Dr. Mary Bennett, who saw him in the early morn of Aug. 27th.

I saw him about 7 a. m. The mother said he was taken with the hoarseness the evening before. The breathing was quite difficult—but not especially distressing—there was some cough, which was harsh and dry. It was ordered that he be given 1-10 gr. turpeth mineral every hour, and that he continually inhale from the steam atomizer a solution of glycerine. I saw him again about 1 p. m., breathing a little more labored. Had taken the medicine, but refused to inhale the vapor, continued the turpeth mineral, and gave half a teaspoonful of glycerine every hour.

I saw him again at 5 p. m., when I met Dr. Harcourt, for whom the parents had sent. The medicine had produced some vomiting. The respiration had now become quite difficult, the obstruction in the larynx having steadily increased during the 10 hours that I had observed the case. The sternal spaces were depressed markedly at every effort of respiration. And although the boy had not been really so sick but that he played with the other children until bed time the evening before, both tonsils and a large part of the pharynx, were covered with a dense diphtheritic deposit. There seemed to be no reasonable expectation that the obstruction could clear up in time to save the child. I advised an operation, which was consented to, and about 6 p. m., with the assistance of Drs. Wadsworth and Harcourt, I opened the trachea, and introduced a tube. Chloroform was given. Respiration was at once free and easy, the boy slept for an hour or two quietly. He utterly refused to take medicine, or food even, for fear that there was medicine in it. He would take glycerine, however, and was given half a teaspoonful every half hour, and allowed to take anything he would. After the second day he took a sugar coated pill of quinine, one grain, three times a day for four or five days. He would not inhale vapor, nor was it practicable to keep the room at an even and elevated temperature. The surroundings of the patient were squalid, and the air of the apartment unwholesome. Yet the boy recovered steadily without an unpleasant symptom, the diphtheritic throat getting well rapidly, and the larynx clearing up so that the tube was removed on the eleventh day. The night following the removal of the tube, there was some embarrassment of respiration. I saw him, but did not deem it necessary to replace the tube, as the derangement was due to moderate spasm. I gave a dose of paregoric, and he had no further trouble. In this case we had extensive diphtheritic exudation, and early involvement of the larynx threatening early death from obstruction, with no severe general disturbance, the child not feeling sick enough any of the time to keep him in bed. There was very little tracheal, and really no bronchial, inflammation. There was no difficulty at all in managing the tubes. This case illustrates how satisfactorily a bad case may be thus treated, and how little trouble it may occasion, even under the most unpromising circumstances.

Case VII.—Nov. 12th, 1876. Rossene, aged 2 years, 2 months, a patient of Dr. White; ill of diphtheria for several days. I was called about midnight by Dr. Jno. Bartlett, who had been called in the emergency. There was severe laryngeal complication which had existed for two days. It was apparent that the child could live but a few hours without relief. I advised the operation, and with the assistance of Drs. Bartlett and Simpson, I opened the trachea and introduced a tube—chloroform was given. When the trachea was opened there welled up into the wound about two teaspoonfuls of muco-pus. There was also discharged through the tube by cough some bloody muco-pus. The child breathed easily but too rapidly to be a promising case. The discharge of muco-pus from the trachea through the wound, and rapid breathing after introduction of the tube and first clearing of the same, are unpromising evidences. The temperature was raised to about 75° and the air made moist by steam from boiling water, also glycerine solution was inhaled from the steam atomizer, and the child fed at frequent intervals. The patient was quiet but the breathing became more and more rapid and patient more exhausted. Death ensued about eighteen hours after the operation. The cause, in my opinion, was an inflammatory change which had begun previous to the operation, in a large portion of lung.

CASE VIII.—On the morning of February 5th, 1877, I was called to see Charley Kennedy, aged 3½ years, a patient of Dr. H. M. Lyman. He had been sick of diphtheria for about a week; for several days had been somewhat hoarse, and markedly more so at night. During the past night respiration had been continuously difficult, and no easier during the morning, as had been the case on other days. The evidences of laryngeal obstruction were well marked. Depression of all the soft spaces * about the chest. Remedies for relief had been used without benefit. I advised tracheotomy, and at 1 P. M., with the assistance of Drs. Lyman and Parkes, I opened the trachea and introudced a tube, using a small quantity of chloroform. A quantity of muco-pus, with shreds of membrane, were discharged as soon as the trachea was opened. The breathing remained hurried and pulse quick after the tube was introduced. He slept for a time after the operation. The accumulation of mucus and membrane in the tube and trachea necessitated its frequent clearing and the introduction of a cotton swab, into the trachea to clear it. Aside from this annoyance, the breathing was comparatively easy, yet the patient steadily failed, although slowly, for two days, dying the

* The terms sternal, soft and interspaces are used to indicate the supra- and infra-sternal, supra-clavicular and inter-costal spaces; also the abdominal wall at the border of the ribs.

fourth day of asthenia and accumulation of dry mucus and membrane in the trachea below the tube.

Case IX.—Evening of April 1st, 1877. Saw Maude Stanley, aged 3 years, with Drs. Bartlett and Daniels. She had been ill of diphtheria for several days; evidences of the larynx becoming involved since morning. The respiration was difficult, but not so urgent as to demand an operation. It was decided to have her breathe steam from slaking lime, which was carried out faithfully for several hours, but the respiration became steadily more difficult, so that by early morning it was evident that if she was to be saved, it must be by tracheotomy. About 5 a. m. of the 2d, with the assistance of Drs. Bartlett and Daniels, I opened the trachea, using chloroform as an anæsthetic; after the tube was introduced respiration was easy; the tube had to be cleaned often of mucus and shreds of membrane. Several times during the first few days the respiration became very much embarrassed by accumulation of shreds of membrane and dry mucus at the lower end of the tube and below. It was expelled by being moistened with glycerine by means of the swab. Dr. Daniels was living in the same house, and was at hand to render timely and efficient assistance in the care of the case. The child utterly refused to take food of any kind. Injections of beef tea were used, and continued for many days, every four or five hours, and the abdomen and chest bathed with cod liver oil twice a day. The temperature of the room was kept from 75° to 80°, and moist, by means of boiling water and a steam atomizer. On the fifth day the wound had become so much ulcerated that it was necessary to remove the tube, to be enabled to make applications to it. The opening had become so large, and the edges so infiltrated, that it remained patulous until the larynx became clear enough for respiration, which was not the case until after two full weeks. There was applied to the wound a solution of carbolic acid (grs. 8 to the ounce) glycerine and water (one part of the former to three of the latter), by means of the atomizer, the spray playing upon the wound for two or three minutes every hour for several days. There was but little increase of ulceration after the removal of the tube, and it soon began to improve.

The history of the case in brief is, that after about 10 or 12

days she began to take food, the wound gradually closed, the larynx cleared so that at about the end of the second week air could pass, and she began to whisper audibly. Her condition steadily improved in every respect, and after a rather prolonged convalescence, she entirely recovered.

CASE X.—Saw Willie Kerfoot, aged 10 years, with Dr. Jno. Bartlett, June 21st. He was taken sick on the 17th with diphtheria, and seemed to be profoundly affected. The tonsils were very much enlarged, quite filling the faucial opening. They were covered completely with a thick diphtheritic membrane, which extended upon the contiguous soft tissues, also into the posterior nares. The breathing was somewhat embarrassed from the occluded condition of the faucial opening. 22d, patient had pretty steadily failed. The nasal passages were more generally involved, and there was slight laryngeal invasion, which become more and more pronounced toward evening. Early in the evening, Drs. Byford and Mannheimer saw the patient, with Dr. Bartlett and myself. It was thought from the general condition, that tracheotomy offered so little, if anything, in the way of relief, that it was not advised, the feeling being that while it was not unwarrantable, it was not worth while to urge it. It seemed that the little boy would live but an hour or so. About three hours later, Dr. H. A. Johnson saw the patient, and, while tracheotomy promised so little, he very strongly urged the operation on the ground that it would be a relief to respiration for the rest of the time the boy might live, even if it proved to be of no other benefit; that the patient was no worse off with, than without it, and that it was the only thing which did offer any chance of relief or escape. About 1:30 a. m., 23d, with the assistance of Drs. Bartlett and Johnson, I opened the trachea without anæsthetic. A large quantity of muco-pus was discharged through the wound as soon as the trachea was opened. A tube was introduced, and the breathing became comparatively easy, but hurried. The pulse remained feeble and very rapid. The patient steadily sank, and died of exhaustion or asthenia about ten hours after the operation.

In this case tracheotomy prolonged life a few hours, and rendered the termination less distressing. It was a case of malignant diphtheria, and on account of the profound diphtheritic infection, tracheotomy promised but very little if any benefit. Trousseau advises against operating in malignant cases. Early amputation of chronically enlarged tonsils we believe should be resorted to when they are attacked by diphtheria, and from swelling and coating with false membrane, they encroach sufficiently upon the faucial opening to interfere with respiration, especially if the nasal cavities are also implicated. In a similar case I would advise early amputation of the enlarged tonsils. In this case certainly, the very large tonsils becoming swollen and covered by thick membrane, and the nasal cavities being attacked also, there was embarrassment of respiration before the larynx became involved.

Case XI.—Aug. 27th, 1877. I saw with Dr. Jno. Bartlett, about 10 p. m., Edward Cross, aged 7 years, ill of diphtheria about ten days. He commenced breathing hoarsely four days before, and had been better and worse up to date, when he had become steadily worse. Respiration was labored and noisy—supra- and infra-sternal and supra-clavicular, and intercostal spaces depressed markedly during every effort of inspiration. There was short ringing cough, expectoration of mucus tinged with blood, and diphtheritic deposit on and behind the tonsils. The little boy was well nourished, and not markedly reduced by the disease. The usual remedies had been resorted to, the laryngeal obstruction had, during the last twelve hours, become markedly more and more pronounced, so much so, that it was evident that unless it could be overcome, death would result in a few hours. Emetics had been used without relief. Tracheotomy was advised, and consented to by the parents, and with the assistance of Drs. Bartlett and Hooper, I opened the trachea and introduced a tube, using chloroform for an anæsthetic. When the trachea was opened, there was ejected some shreds of membrane, together with a little blood and mucus. He slept quietly, and breathed very easily after the operation. During the 28th he was very comfortable, being troubled but little with cough or clogging of the tube with mucus. Several times during the day and night pieces of membrane were discharged through the tube. He took a reasonable amount of milk and broth for food. A fine papular eruption showed itself on the forehead and in the edge of the hair. 29th, patient had not slept well during the night; did not feel quite as comfortable; cough rather more severe, some membrane, mucus and a little blood discharged through the tube; he had taken his food well; the neck and tissues about the angles of the jaw were swollen a little more; the eruption had extended to the face. 30th, morning—slept fairly during the night, and has taken food well, but is less comfortable; face and neck more swollen; eruption has extended over all of the face, ears, neck, and somewhat on chest and back; coughs and expectorates less; wound a little more tender; complains of soreness behind the sternum. Evening—Has been more comfortable the latter part of the day; had a long sleep; breathes very quietly; but little mucus being discharged; eruption gradually extending. 31st, morning—He slept quietly most of the night, having been annoyed but little by cough; face and neck less swollen; eruption fading from face; breathing very easy and free: he is to-day much more comfortable and really better. Sept. 1st, continues better. 2d, still better, eruption fading. 3d, better. 4th, morning, the tube became foul with mucus and pus adhering to the neck plate, tapes and tissues about the wound; it was removed and a clean one introduced—a Fuller bi-valve. Everything went on well until the night of the 7th, when about 11 o'clock the inner tube was removed for cleansing, and on an attempt at re-introduction there was found to be some obstruction, which caused some impediment to respiration. I saw the patient soon after, when I found that the granulations, which were very abundant about the wound, had fallen into the openings in the tube, filling it so that the inner one could not be replaced. I removed the tube and introduced one without any opening; respiration was resumed as usual. After several days this tube was changed for one with a fenestrum. One day, when the inner tube had been removed for cleaning it, could not be reinserted, and on inspection the tube was found nearly filled with a mass of granulations which had fallen into the fenestrum. The tube was forcibly removed, bringing away with it the cluster of granulations. After this the granulations were cauterized with nitrate of silver or carbolic acid, daily or every other day, and the repressed breathing and voice steadily became more and more clear, when, on the 10th October, the tube was removed and the wound allowed to close. This was fully accomplished at the end of forty-eight hours.

Case XII.—October 9th, 1877, at 4.30 p. m., I saw with Dr. Matthei, Willie McCoy, aged 7 years and 2 months, who had been ill of what seemed to be an ordinary sore throat, with some hoarseness for three or four days. He had always been subject to attacks of spasmodic croup. This had persisted, and the breathing steadily became more embarrassed. Dr. M. was called this morning, and from the means used, the breathing had been quiet during the day. The fauces were somewhat tumid and reddened, but no patches of membrane were detected; respiration was embarrassed, but not urgently so; the intercostal spaces of the thorax were moderately depressed. A continuance of the remedies was advised, with the addition of about 8 gr. bromide of potassium, a poultice to the neck, and the use of the steam atomizer. 10th, at 9 a. m., I saw the patient again. The breathing had become steadily more and more difficult, so that he was showing decided evidences of laryngeal obstruction, which would prove fatal in course of a few hours if left to itself. All of the thoracic spaces were markedly depressed, and the surface had begun to be cyanosed. Tracheotomy was advised, and consented to by the parents. Chloroform was given, and, with the aid of Dr. M., I opened the trachea, about 10 a. m. The patient became a good deal depressed during the operation, respiration being very feeble before its completion. A large piece of membrane, with mucus, was expelled through the wound. When the tube was introduced some bloody mucus was also discharged, and the respiration became very good. The patient drank a reasonable quantity of milk, expectorated fairly, rested easily, and seemed to be doing as well as could be expected until the evening of the 12th. The neck about the wound had become inflamed and swollen; respiration became difficult; no expectoration. At about 11 p. m., when I saw him, there was evident obstruction below the tube, it being clear; a swab saturated with glycerine and passed down the trachea revealed no obstruction, yet there was persistent difficulty in breathing. I gave a teaspoonful of paregoric, oiled the inflamed neck, and caused him to inhale from the atomizer steam from lime-water, glycerine and a little carbolic acid. The breathing was a little easier at times, but became more labored and difficult during the latter part of the night and the morning of the 13th, when it seemed that the boy could live but a short time. He was quite cyanosed, and struggled for breath, tossing about the bed, or clinging to the attendants wildly for relief. So he continued during the forenoon and till about 2 p. m., at times apparently ready to die; but about this time there was expelled a quantity of membrane, and some plugs of hardened mucus, which gave him great relief. He was much exhausted, but now slept and rested. In the evening the breathing became somewhat difficult. I removed the tubes and applied glycerine to the trachea by means of a catheter, and persistently kept up the inhalation of a solution of chlorate of potassa and glycerine in water, which soon caused the expulsion or discharge of bits of dried mucus and some pieces of membrane, which rendered the breathing comparatively easy. The wound remained patulous, so the tube was left out. The wound and inflamed neck were lubricated frequently with cod-liver oil—steam to be inhaled freely, an oiled silk jacket put around the chest, a poultice over the sternum, and an injection of beef tea and two grains of quinine to be repeated every three or four hours. During the latter part of the night, and all of the 14th, and the following night, he was pretty comfortable—occasionally a piece of membrane or hardened mucus would be expelled, and there was expelled from the tube, by coughing, a fair amount of muco-pus. During the morning of the 15th the breathing became somewhat difficult. When I saw him, the opening in the trachea had become contracted sufficiently to offer a little impediment to respiration; the inflammation had subsided markedly. I cleared the edges of the wound of adherent crusts, and introduced some glycerine into the trachea. There was expelled quite a quantity of secretion. I then introduced a Fuller bivalve tube. He now breathed freely, drank some tea and milk, some of which came out through the tube. I gave four drops of deod. tincture of opium, and continued the other treatment. It had not been carried out very well during the night. He breathed reasonably easy during the day, slept somewhat, but had swallowed very little food. The beef tea injections had been given faithfully. At 5 p. m. I removed the tube. There was free discharge of muco-pus from the trachea. He breathed somewhat through the larynx, and could speak in a very audible whisper. Visited him at 11 p. m.; found him very comfortable. When asleep the breathing was labored, yet the chest filled well. As a precaution, I introduced a tube to be left in during the night, and ordered a continuation of the treatment. 16th, 8 a. m, patient slept and rested very well during the night, looking brighter and happier than at any time before; says he feels better; breathes very easily. Tube was removed about 4 a. m. 9 p. m, tube has been out all day; has breathed without difficulty. There has been quite abundant secretion during the afternoon and evening; added a little carbolic acid and tincture of myrrh to the inhalation. 17th, morning, patient has had a very comfortable night, and feels better, is very weak, but begins to take a little more food; fluids occasionally pass by way of the wound; inflammation of neck and wound disappearing. Evening, has had a very comfortable day. 18th, morning, every way comfortable; also at evening. 19th, better; takes more food. 20th, respiration perfectly easy; more air passes the larynx; wound cleaning off and patulous. 22d, 3:30 p. m, has had quite a high fever since about 9 a. m., pulse about 130, vomited once; ordered grs. v. sulphite of soda and one drop of tincture of aconite root every three or four hours. 23d, 9:30, fever subsided, slept well during the night; has had three loose passages this morning, breathes easily, mostly through the larynx. Continued the medicine of yesterday every eight hours, and ordereed 1 gr. quinine every eight hours. 5 p. m, has been pretty comfortable during the day. 24th, slept fairly through the night, complains of pain in the limbs; ordered 4 drops of tincture of iron and 2 drops of tincture nux vomica every 6 hours. 25th, has complained of a good deal of pain in his limbs and about the chest; slept only fairly, breathing somewhat hurried, right side of chest seems slightly dull on percussion, pulse about 120; takes milk and coffee. I continued the iron mixture, and ordered a poultice to the right side of the chest. 5 p. m., has been uncomfortable all day, pain in legs, breathing labored and rapid, pulse 120 and a little irregular; has taken but a moderate quantity of food during the day. Ordered the elixir of cinchona ℥i, and quinia grs. i, every 4 hours; also 15 grs. of bromide of sodium and 3 drops of tr. opii deod., twice during the night. 26th, has failed perceptibly since last evening. Died about noon of pneumonia, the 16th day after the operation, and 10 days after the tube was removed, having become quite anemic and emaciated. He had neuralgic pains, albuminous urine, loathing for food, and paralysis, or a want of sensitiveness, of the glottis, (fluids being allowed to enter the larynx,) showing the general and secondary manifestations of diphtheria. This case is of unusual interest, for from the evidences at first, and from the amount of membrane (for there was a great deal discharged at the time of operation and afterward), it may have been called a case of membranous croup, as distinguished from diphtheritic croup. But we believe it, without doubt, to have been a case of diphtheria. In this case the operation was a success, for it rescued the patient from dying of laryingeal obstruction, death not occurring until several days after the larynx had become clear.

While the operation in this case should be included in the list of successes, it having accomplished its object as a remedial measure, we class it with those marked unsuccessful, because the patient did not recover.

Case XIII.—Oct. 12th, at 4 p. m. I was called, with Dr. D. S. Root, to see Baby Wasserman—a patient aged 2 years and 2 months. He commenced breathing hoarsely, on the morning of the 11th. The hoarseness had steadily increased and breathing become more and more difficult, until now there is only a faint whisper and the soft spaces about the chest are all markedly depressed with every attempt at inspiration, the front of the chest also being markedly depressed during the act; pulse very rapid and the surface becoming cyanosed. Tracheotomy was advised, and with the assistance of Drs. Root and Fisher I opened the trachea at the superior point, after giving a little ether. The isthmus of the thyroid gland was very large; it was separated and held forcibly downward to make the tracheal opening and to introduce the tube. During the latter part of the operation the patient became very low, quite thoroughly cyanosed and very nearly ceased to breathe. Some delay was experienced in introducing the tube, but it was finally accomplished easily by first passing into the trachea a soft catheter and slipping the tube over it. As soon as the tube entered the trachea the catheter was withdrawn. There was immediately discharged some blood and mucus. After this the child breathed with ease and slept quietly, it was put to bed and a hot bottle placed near the feet. He rested very well during the night and was quite comfortable the next day, the 13th, yet was a little feverish and breathed rather rapidly. During the night following there was more cough and discharge of thin muco-pus. He had a slight chill on the morning of the 14th. Breathing hurried, cough annoying, neck swollen, thyroid gland inflamed, and there was diarrhœa. It seemed evident that pneumonia or bronchitis had developed. The tube was changed, chest rubbed with camphorated oil and an oiled silk jacket put on—during the day the breathing became more hurried, pulse faster, and strength failed. The child died about 4:30 p. m., of broncho-pneumonia.

Case XIV.—Monday, October 22d, 8 p. m., I was called to see Mary Donehue, aged three years 11 months, who had been ill since the evening before. During Saturday night she had a slight attack of difficult breathing, but it had passed away before morning. Coughed a little, and was slightly hoarse during the day, but at evening she became quite hoarse, and breathed with some difficulty during the night. In the morning, Dr. Constance saw her, and used the ordinary relaxing remedies. The breathing became steadily more difficult, and she could only whisper. In the evening, Dr. F. L. Wadsworth saw her, who called me with reference to tracheotomy.

The child was thoroughly exhausted from the prolonged struggle to breathe. All of the interspaces of the chest were markedly depressed at every effort of inspiration, which was very harsh and noisy ; could only whisper, coughed occasionally, and was wet with copious perspiration.

I advised tracheotomy, and with the assistance of Drs. Wadsworth and Constance, opened the trachea and introduced a tube, giving chloroform for an anæsthetic. There was no delay during the operation, and but trifling hemorrhage. A small quantity of mucus was coughed up when the trachea was first opened. She was to be kept warm and the air moist by steam. She slept and breathed with perfect comfort directly after the operation, and remained very comfortable during the night. 23d, 8:15 a. m., found patient comfortable and breathing easily ; but little mucus had been coughed up during the night ; was a little feverish, and pulse quite rapid. Ordered 2½ grains sulphite of soda and half a drop of tincture aconite every 2 hours and a half.

8 p. m. She has passed the day quite comfortably ; had one rather hard spell of coughing ; has drunk milk, and slept some ; medicines continued ; the steam atomizer to be used half of the time during the night, at half hour intervals.

24th, morning.—Has slept fairly and been quite comfortable. Evening.—Has seemed not quite as comfortable during the afternoon ; breathes easily ; raises more mucus ; has a bright red lichenoid eruption over most of the body and limbs since yesterday morning.

25th.—Passed a good night, and is very comfortable. Ordered 3 drops tincture ferri chlor. every 4 hours. Evening.—Has been very comfortable all of the day.

26th.—Slept very well most of the night ; is in every way comfortable this morning. Evening.—Comfortable all day.

27th.—Very comfortable ; no fever ; coughs but little ; takes more food.

From this date to November 12th, she continued well, the larynx clearing up, so that the voice and breathing became easy and natural, when the tube was permanently removed. The tube could have been removed a few days earlier with probable safety, but on trial the respiration seemed a little rough, and as the tube was a source of no annoyance to the neck and trachea, it was allowed to remain.

CASE XV.—I was called by Dr. H. M. Lyman, Dec. 5th, to see George Healy, aged 7 years, who had been sick of pharyngeal diphtheria for several days. The larynx became implicated on the 4th, and the difficulty in breathing had steadily increased since then. When I saw him the evidences of laryngeal obstruction were well marked by labored effort in respiration and the sinking in of the interspaces of the chest ; the pulse was rapid and feeble, the surface cyanosed. Although the patient was in a condition unpromising for operation, I advised tracheotomy ; and with the assistance of Drs. Lyman and Hempstead opened the trachea, and after removal of some shreds of loose membrane introduced a tube, using a very little coloroform. Respiration was perfectly easy through the tube ; the blueness of the lips soon passed away, but there was a good deal of depression, the pulse was not felt at the right wrist, and but feebly at the left. He was put to bed and hot irons placed about the limbs. He slept quietly. In about half an hour the pulse had become perceptibly stronger and the surface warm. A little bloody mucus had been discharged through the tube. The temperature was raised to 75° and the air made moist by steam from an atomizer. He slept for a couple of hours ; reaction became well established, and while the breathing was easy it remained too rapid, as well as the pulse. He took a little food and remained very comfortable until the latter part of the night, when the breathing became noisy and labored, which difficulty steadily increased, the patient dying about 5 p. m. of the 6th, of suffocation, from what seemed to be accumulation of membrane and dry mucus in the trachea or bronchi, together with congestion of the lungs. This case illustrates well what is likely to follow the operation where it is delayed to the third stage of the disease. There will be very good reaction and very hopeful promise for 12 to 24 hours ; then death results from some lung complication that has had its origin in the injury which that organ has suffered from the prolonged struggle and congestion before the operation. It is always ominous to have the pulse and respiration continue rapid for some hours following tracheotomy, and hopeful if they become gradually slower.

Of these patients, the ages ranged from 2 to 10 years. The number of cases operated upon between

| | | | | | | |
|---|---|---|---|---|---|---|
| 2 | and | 3 | years | was 3. | Recovered, | 0. |
| 3 | " | 4 | " | " 4. | " | 2. |
| 4 | " | 5 | " | " 2. | " | 2. |
| 5 | " | 6 | " | " 2. | " | 1. |
| 7 | " | 8 | " | " 3. | " | 1. |
| 9 | " | 10 | " | " 1. | " | 0. |
| | | | | — | | — |
| | | | | 15. | | 6. |

The tube was worn in the cases of recovery 44, 21, 5, 16, 120 and 11 days, respectively. In the case in which the tube was worn 44 days, its removal was delayed by granulations at the tracheal border of the wound; and in the one in which it was worn 120 days, the delay was occasioned from paralysis of the larynx or glottis; and in the case where the tube was worn only 5 days, it was not removed because the larynx was clear at that time, but on account of ulceration of the edges of the wound, the sides of which were infiltrated so that the opening remained patulous until the breathing could be carried on through the larynx. It was not until after two full weeks that it had become thus clear. In case XII, the edges of the wound ulcerated, and became infiltrated with inflammatory exudation, so that it remained open for respiration for several days after the tube was removed, before the larynx became clear, which was the case several days before death. Those who did not recover, died in from 10 hours to 16 days, most of them within 48 hours.

From our experience in the above cases, we are led to the following conclusions:

1st.—That the so-called membranous croup, as we see it here, and diphtheritic croup, are all cases of the same disease; all are diphtheritic. In all of these cases, the children had been ill of pharyngeal diphtheria, or evidences of the disease showed themselves later, and in most of them diphtheria attacked other children of the family, except it be in cases I and XIV. None of the other children in these families were ill, I think; yet from the condition of the wound and general condition of case No. 1, I was satisfied that he was affected with diphtheria. In case XIV, the evidences were not so clear, but I believe it was of the same character, confined to the larynx. If not identical, they are so practically, as regards treatment, neither requiring treatment which is not applicable to the other. No antiphlogistics are useful for the one or the other. We believe that the essential difference is only in the place where the disease or membrane first locates itself. If in the pharynx, it it usually seen before the larynx becomes affected. If in the larynx, the swelling and deposit of membrane may in a very short time obstruct it sufficiently to destroy the patient even in a few hours. This can be readily understood when we remember how much the tonsils may be swollen, and how much membrane is sometimes seen very soon after the child is taken ill, if it is looked for, and this, too, when the child does not appear to be very sick. If this condition were to be located at the glottis, it would compromise life early. In some cases, the disease is first located in the trachea, and membrane is formed there before the larynx becomes involved, for in some of the cases the larynx had been implicated but a short time before the operation. But when the trachea was opened, loose membrane was found there, and was expelled or removed with the forceps, and yet the larynx was blocked up with adherent membrane, which was not separated for several days.

2d.—That tracheotomy should be resorted to in all cases where death is threatened by suffocation from obstruction in the larynx, and as soon as the breathing has become insufficient to sustain the vital powers. This will be evident from the difficulty with which respiration is performed, from exhaustion from want of rest, from the severe muscular efforts to respire, the restlessness and the anxious expression. The degree of obstruction will be measured by the extent of labored movements of the chest and by the amount of depression of the inter-spaces of the chest on inspiration. If the obstruction has been quite acute, the respiratory efforts will be great, but if it has been developed gradually, they will be less laborious. Some preparation of opium or the bromides should be given to overcome whatever spasm may exist, steam inhaled—which may be medicated according to one's notions respecting the properties of various remedies in softening or dissolving membrane, or soothing the inflamed parts : heat and moisture should be applied to the front of the neck, and in some cases a prompt, but not depressing, emetic used. If these procure no relief, the operation is indicated. If there has been steadily increased difficulty in breathing for a variable time, until there is obstruction sufficient to prevent the entrance of enough air to the lungs to satisfy the demand, the operation ought to be made—and this too, before the patient is exhausted.

It should be resorted to during the so-called second stage of the disease; while there is yet a reasonable amount of strength; at this time there is a great deal more to be hoped for from the operation. If it is delayed until the third stage, or until the pulse has become feeble and frequent or gone at the wrist, the surface cold and blue, there will have been prolonged congestion of the arterial side of the lungs and the right side of the heart; which, with the greater degree of exhaustion, will compromise the chances of success very much, and there will be more likelihood of a bronchitis or pneumonia being developed—which will pretty certainly prove fatal. In these cases there will usually be temporary improvement, but the lung complication which had already begun, will become estabtablished and carry off the patient.

3d.—That it is best, in the majority of cases, to use an anæsthetic. Respiration is usually rendered somewhat easier from whatever spasm of the larynx may exist, being controlled, the child also is saved from the pain and fright of the operation. Ether no doubt is the safer anæsthetic to use, and is to be preferred, although we have used chloroform in most of our cases.

4th.—That the operation should be made slowly and by carefully dissecting down upon the trachea, avoiding all vessels which can be seen and protected from division, and unless there is some urgent reason for opening the trachea before all bleeding has ceased, this should first be controlled. The trachea may be opened above or below the isthmus of the thyroid gland, whichever is most convenient. In some cases the gland will not be noticed during the operation, in others it will be a source of annoyance from its size, but it can be held out of the way with a hook or tenaculum.

5th.—That a tube should be used. It will keep the wound open better than any thing else, and cause as little irritation. It should be as large as the trachea will admit. The opening should be not less than about one-fourth of an inch, for the trachea of a child somewhat less than two years, will admit readily one of that size. It should be double, and may have a moveable neck plate, or not; there are advantages in both kinds; the same may be said of fenestra; it will be found best in one case to use a tube with fenestrum, and in another one without. The tube should be worn until the larynx has become clear enough to allow respiration to be performed easily. This can be ascertained by corking the tube, and for this a tube with fenestrum is best. The tube may be removed for trial, to be replaced if respiration becomes difficult.

Should the wound ulcerate, the tube must be removed, if the wound will remain open, so as to allow easy breathing. It may be left out for a part or all of the day, or wholly, as the case will allow. Almost any stimulating application may be used for the wound, if it is unhealthy. If it is not, nothing need be done. The inner tube will have to be cleaned of mucus and other secretions sufficiently often to allow free passage of air; this may be at intervals of from ten minutes to an hour or more. It must not be left out for a longer time than suffices for washing and clearing, so as not to allow the outer tube to become obstructed, for if it does, when the inner tube is replaced, the accumulation will be carried into the trachea, or will adhere to the end of the inner tube; in either case it would be likely to give trouble by interfering with respiration.

6th.—That the temperature of the room should be not less than 75°, moist, and free from currents of air. The air should, for the first few days at least, be kept thoroughly saturated with steam from boiling water, or from the steam atomizer. The vapor may be medicated to suit the various fancies respecting the properties of medicaments. We have used mostly a solution of glycerine, about one-fifth or one-sixth part glycerine in plain water, a little chlorate of potass, or carbolic acid, may be added. And a loose woolen scarf should be kept about the neck, that the air may be respired through it. The object of the vapor is to secure a moist condition of the trachea and bronchi, to prevent the accumulation of dry mucus and shreds of membrane below the tube. The atomizer may be allowed to play directly upon the neck for a short time, at intervals, if it seems necessary to have additional moisture, but it will be too cold and wet to be kept thus, much of the time. It will not do to have only a semblance of moisture, the air must be kept saturated.

7th.—That the patient must be sustained by such food as is most acceptable or would seem most appropriate for each individual case. Occasionally there will be utter loathing of food, or an absolute impossibility to induce the child to swallow it, as in case IX. It will then be necessary to resort to artificial means of feeding. Such remedies as may seem indicated should be given. The general and even local treatment of the disease should not be omitted after the operation. If there should be any indications of ensuing lung complication, the chest ought to be enveloped with a hot poultice, or a rubber or oiled silk jacket applied, the surface of the chest first irritated by a mustard paste or rubbed with kerosene or camphorated oil, and other appropriate remedies used. If at any time the patient is restless or wakeful, it had better be kept reasonably quiet by the use of some opiate or one of the bromides; and when the child can breathe through the larynx for a prolonged time without discomfort and can talk again, the tube may be removed. The edges of the wound will fall together and become adherent in from one to two days, requiring no dressing. If after the tube has been removed the respiration should become difficult, the tube must be replaced by forcibly dilating the opening, or even enlarging it by cutting, if that is necessary. There may be, during the first night after the removal of the tube, some difficulty in breathing from spasm of the larynx, which will usually be relieved by an anodyne, such as a full dose of paregoric. If it does not give trouble the first night, the patient is quite secure from this kind of annoyance.

In none of these recovered cases, has there been any impairment of the voice.

3 Washington Place, Chicago.

